# Global Lysine Acetylome Analysis of Desiccated Somatic Embryos of *Picea asperata*

**DOI:** 10.3389/fpls.2016.01927

**Published:** 2016-12-23

**Authors:** Yan Xia, Danlong Jing, Lisheng Kong, Jianwei Zhang, Fangqun OuYang, Hanguo Zhang, Junhui Wang, Shougong Zhang

**Affiliations:** ^1^State Key Laboratory of Tree Genetics and Breeding, Key Laboratory of Tree Breeding and Cultivation of State Forestry Administration, Research Institute of Forestry, Chinese Academy of ForestryBeijing, China; ^2^Centre for Forest Biology, Department of Biology, University of VictoriaVictoria, BC, Canada; ^3^State Key Laboratory of Tree Genetics and Breeding, Northeast Forestry UniversityHarbin, China

**Keywords:** lysine acetylome, conifer, somatic embryo, partial desiccation treatment, response to stress

## Abstract

Partial desiccation treatment (PDT) promotes the germination capacity of conifer somatic embryos. Lysine acetylation (LysAc) is a dynamic and reversible post-translational modification that plays a key role in many biological processes including metabolic pathways and stress response. To investigate the functional impact of LysAc in the response of *Picea asperata* somatic embryos to PDT, we performed a global lysine acetylome analysis. Here, combining antibody-based affinity enrichment and high-resolution mass spectrometry, we identified and validated 1079 acetylation sites in 556 acetylated proteins from *P. asperata* somatic embryos during PDT. These data represent a novel large-scale dataset of lysine-acetylated proteins from the conifer family. Intensive bioinformatics analysis of the Gene Ontology of molecular functions demonstrated that lysine-acetylated proteins were mainly associated with binding, catalytic activities, and structural molecular activities. Functional characterization of the acetylated proteins revealed that in the desiccated somatic embryos, LysAc is mainly involved in the response to stress and central metabolism. Accordingly, the majority of these interacting proteins were also highly enriched in ribosome, proteasome, spliceosome, and carbon metabolism clusters. This work provides the most comprehensive profile of LysAc for a coniferous species obtained to date and facilitates the systematic study of the physiological role of LysAc in desiccated somatic embryos of *P. asperata*.

## Introduction

Lysine acetylation (LysAc) is a ubiquitous, reversible and highly conserved post-translational modification (PTM) of both histones and non-histone proteins of prokaryotes and eukaryotes (Kim et al., 2006; Choudhary et al., 2009; Weinert et al., 2013). This modification represents an effective approach for transcriptional regulation and modulates diverse properties of proteins, such as protein activity and localization and protein-nucleic acid and protein-protein interactions (Arif et al., 2010). Since the first identification of LysAc in histones (Phillips, 1963), this modification has become well known for its primary functions in relaxing chromatin structure, weakening histone-DNA interactions, and altering histone-histone interactions (Strahl and Allis, 2000; Eberharter and Becker, 2002). However, histones are not the only proteins that are highly regulated by LysAc. Thirty-four years after the first report of this modification, LysAc was identified in the non-histone protein p53 (Gu and Roeder, 1997), greatly extending the functional scope of LysAc research. Furthermore, mechanistic studies have indicated that a large number of lysine-acetylated proteins impact various nuclear and cellular processes, including chromatin dynamics, transcriptional regulation, and metabolic pathways (Glozak et al., 2005; Yang and Seto, 2008; Choudhary et al., 2009). Notably, functional annotation demonstrated that LysAc is mainly associated with regulation of enzymatic activities, metabolic pathways, and stress responses (Tootle and Rebay, 2005; Zhang et al., 2009; Liu et al., 2016).

The first system-wide analysis of LysAc was conducted in HeLa cells and mouse liver mitochondria (Kim et al., 2006). Since that time, the number of LysAc sites identified in proteins has continuously increased in mammalian cells and bacteria (Choudhary et al., 2009). More recently, with the advantages of anti-acetyllysine-based enrichment and high-resolution mass spectrometry (MS), lysine acetylome studies have been begun to be performed in plants. The first systematic studies of LysAc demonstrated that during *Arabidopsis thaliana* development, LysAc modification participates in the regulation of central metabolic enzymes (Finkemeier et al., 2011; Wu et al., 2011). Thereafter, analyses of the lysine acetylomes of *Vitis vinifera* (Melo-Braga et al., 2012), *Oryza sativa* (Nallamilli et al., 2014; He et al., 2016; Xiong et al., 2016), *Pisum sativum* (Smith-Hammond et al., 2014a), *Glycine max* (Smith-Hammond et al., 2014b), *Fragaria ananassa* (Fang et al., 2015), and *Triticum aestivum* (Zhang et al., 2016) revealed that lysine-acetylated proteins are involved in growth and development in many plant species. However, there are no available reports of research on LysAc in coniferous species. Understanding of this modification in conifers is therefore limited.

Spruces are important forest species for timber production in many parts of the world; the fully sequenced genomes of Norway spruce and white spruce provide bioinformatic resources for research on conifer genomics, transcriptomics, and proteomics (Birol et al., 2013; Nystedt et al., 2013). Somatic embryogenesis in conifers was first reported in Norway spruce (Chalupa, 1985; Hakman and von Arnold, 1985) and included embryonic cultures initiation, followed by somatic embryo formation, maturation, and germination. Somatic embryos, which are able to develop into plantlets of identical genotype for afforestation, are ideal materials for studying embryogenic development in conifers. Prior to the embryo germination stage, partial desiccation treatment (PDT) has been used effectively to improve the embryo germination/conversion (De-la-Peña et al., 2015; Yakovlev et al., 2016). PDT is known to involve stress caused by a gradual and limited loss of moisture content (Roberts et al., 1990), which promotes the transformation of these embryos from morphological maturity to physiological maturity (Liao and Juan, 2015). In a previous study, we found that the levels of stress-related proteins increase in somatic embryos of *Picea asperata* Mast. during PDT, and some proteins of photosynthesis metabolism accumulate differentially under low light (Jing et al., 2016). Although LysAc is one of the most prevalent PTMs, it has yet to be investigated as a component of the regulatory mechanism underlying the responses of conifer somatic embryos to PDT. Therefore, a global lysine acetylome analysis of spruce somatic embryos during PDT was investigated.

*P. asperata* is a native spruce widely distributed in China with outstanding wood properties and adaptability (Xia et al., 2016). The highly synchronized somatic embryos from embryogenic cell line 1931 provide identical genotype for proteomics analysis during PDT (Jing et al., 2016). In this study, we used antibody-based affinity enrichment, high-resolution MS, and intensive bioinformatics analysis to comprehensively investigate the lysine acetylome of desiccated embryos of *P. asperata*. By searching the 1360 acetylated peptides identified against the previous proteome dataset of the embryos during PDT, we successfully validated 1079 acetylation sites in 556 acetylated proteins in desiccated embryos. To the best of our knowledge, this is the lysine acetylome study in conifers that has not been reported before and provides a rich resource for functional analysis of lysine-acetylated proteins in conifers to date. Based on the most comprehensive acetylome for *P. asperata* somatic embryos, we now report important lysine-acetylated proteins that are mainly involved in the response to stress and central metabolism during PDT.

## Materials and methods

### Plant materials

Somatic embryos of *P. asperata* were obtained from the embryogenic cell line 1931, which was initiated from an immature zygotic embryo in the National Spruce Germplasm Bank of China (2003DKA21003). According to Xia et al. (2016), the embryos were matured on semi-solid modified Litvay medium (Litvay et al., 1985) supplemented with 0.1% (w/v) casein acid hydrolysate (Sigma, St. Louis, MO, USA), 3% (w/v) sucrose (Beijing Chemical Works, Beijing, PR China), 5% (w/v) polyethylene glycol 4000 (Merck, Darmstadt, Germany), and 0.5% (w/v) gellan gum (Sigma). Prior to autoclaving (121°C, 20 min), the pH was adjusted to 5.8 ± 0.01. At the end of this procedure, filter-sterilized (0.2-μm filter, Pall Corp., Port Washington, NY, USA) L-glutamine (Sigma) and (±)-abscisic acid (Gibco, Grand Island, NY, USA) were added into the autoclaved and cooled medium to the final concentration of 0.05% and 60 μM, respectively. The embryo maturation cultures were maintained in darkness at 24 ± 1°C. After 2 months of culture, mature embryos with well-developed cotyledons were selected and transferred onto two layers dry sterile filter paper (Whatman™ 2, Kent, UK) to undergo PDT under a 16-h photoperiod with a low light density of 15 μmol m^−2^ s^−1^ (LED fluorescent tubes) at 24 ± 1°C for 0 (D0), 7 (D7), 14 (D14), or 21 (D21) days. Finally, the desiccated embryos were collected, and immediately frozen in liquid nitrogen, then stored at −80°C until use.

### Proteomic analysis

#### Protein extraction and western blotting

Proteins were extracted from somatic embryos using the method described previously (Fang et al., 2015; He et al., 2016). Briefly, the embryos were ground in liquid nitrogen. Then, the sample powder was combined with lysis buffer containing 8 M urea, 10 mM dithiothreitol (DTT), 2 mM EDTA, 3 μM TSA, 50 mM NAM and 1% Protease Inhibitor Cocktail Set VI (Calbiochem, Darmstadt, Germany), and sonicated three times on ice using a high-intensity ultrasonic processor (scientz-IID, SCIENTZ GmbH, Ningbo, China). After centrifugation at 20,000 g at 4°C for 10 min, the remaining debris and unbroken cells were discarded. The protein collected in the supernatant was incubated with cold 15% trichloroacetic acid for 2 h at −20°C. The precipitated protein was centrifuged again at 20,000 g at 4°C for 10 min, and the pellet was washed three times using ice-cold acetone to completely remove the trichloroacetic acid. The pellet, which was lyophilized in a SpeedVac (Thermo Fisher Scientific, Inc., Waltham, MA, USA), was finally re-dissolved in buffer (8 M urea, 100 mM NH_4_HCO_3_, pH 8.0).

Protein concentrations were measured using the 2-D Quant kit (GE Healthcare Life Sciences, Waukesha, WI, USA) according to the manufacturer's instructions. The presence of LysAc in desiccated somatic embryos at different stages (PDT 7, 14, and 21 days, that is, D7, D14, and D21), along with non-desiccated somatic embryos (D0) as a control, was demonstrated by western blotting. Briefly, the protein sample was diluted with SDS loading buffer, and 20 μg of protein from each sample was separated *via* 12% SDS-PAGE and electro-blotted onto a polyvinylidene fluoride membranes (Millipore, Bellerica, MA). The blot was then probed with a pan anti-acetyllysine antibody (Catalog No. PTM-101, PTM Biolabs, Inc., Hangzhou, PR China) using a 1:1000 dilution, followed by incubation with a horseradish peroxidase-conjugated secondary antibody (Sigma) at a 1:5000 dilution.

#### Protein trypsin digestion, lysine-acetylated peptide enrichment, and MS analysis

Protein sample (~12 mg) obtained from the somatic embryos on D14 was reduced with 10 mM DTT for 1 h at 37°C, then alkylated using 20 mM iodoacetamide for 45 min in darkness at room temperature. The treated protein was diluted with 100 mM NH_4_CO_3_ to ensure that the urea concentration was < 2 M. Finally, the re-suspended protein was digested with Trypsin/P (Product code V5111, Promega Corp., Madison, WI, USA) at a 1:50 mass ratio of trypsin:protein for the first digestion, conducted overnight at 37°C according to the usage information. For complete digestion, a 1:100 trypsin:protein mass ratio was used, and 4 h of digestion was conducted at 37°C. The digested peptides were lyophilized using the SpeedVac.

To enrich lysine-acetylated peptides, lyophilized peptides were re-dissolved in NETN buffer (100 mM NaCl, 1 mM EDTA, 50 mM Tris-HCl, 0.5% NP-40, pH 8.0) and efficiently recovered through a powerful affinity enrichment procedure using pre-washed pan anti-acetyllysine agarose beads (Catalog No. PTM-104, PTM Biolabs, Inc.), performed at 4°C overnight with gentle shaking (Pan et al., 2014; Fang et al., 2015; He et al., 2016). The supernatant was then removed, and the beads were washed four times with NETN buffer and twice with ddH_2_O. Next, the bound peptides were eluted from the beads using 0.1% trifluoroacetic acid. The released peptides were combined and vacuum-dried again using the SpeedVac. The obtained peptides were finally cleaned with C18 ZipTips (Millipore), referencing the manufacturer's instructions.

Enriched peptides were dissolved in mobile phase A [0.1% formic acid (FA) in 2% acetonitrile (ACN)] and then directly loaded onto a reversed-phase pre-column (C18 trap column, Acclaim PepMap 100, 75 μm × 2 cm, Thermo Fisher Scientific, Inc.). Peptide separation was conducted *via* a reversed-phase C18 analytical column (Acclaim PepMap RSLC, 50 μm × 15 cm, Thermo Fisher Scientific, Inc.). The gradient was comprised of an increase from 6 to 22% mobile phase B (0.1% FA in 98% ACN) over 24 min, followed by 22 to 35% B over 8 min and a climb to 80% B over 5 min, then a hold at 80% B for the last 3 min, all at a fixed flow rate of 300 nl/min in an EASY-nLC 1000 system (Thermo Fisher Scientific, Inc.).

The obtained peptides were subjected to a nanospray ion source, followed by tandem mass spectrometry (MS/MS) in a Q Exactive™ Plus MS (Thermo Fisher Scientific, Inc.) coupled online to the UPLC. Intact peptides and ion fragments were detected in the Orbitrap as described previously (Fang et al., 2015). Based on three additional references (Zhang et al., 2013; Pan et al., 2014; He et al., 2016), we specified a data-dependent procedure that alternated between one MS scan that followed by 20 MS/MS scans for the top 20 loops (scans) with the precursor ion (the full scan) above a threshold ion count of 1 × 10^4^ in the MS survey scan with 15.0 s dynamic exclusion. The electrospray voltage was set at 2.0 kV. The automatic gain control was used to prevent overfilling of the ion trap, and 5 × 10^4^ ions were accumulated to generate the MS/MS spectra. For MS scans, the range of m/z scans was from 350 to 1800 Da.

#### Database search

The results regarding protein and acetylation site identification were processed with MaxQuant software using the integrated Andromeda search engine (version 1.4.2) (Cox and Mann, 2008; Cox et al., 2009). Tandem mass spectra were searched against the Congenie database (26,437 sequences on November 15, 2015, ftp://plantgenie.org/Data/ConGenIE/) concatenated with a reverse decoy database. Trypsin/P was set as the cleavage enzyme and the search allowed up to four missing cleavages, five modifications per peptide and five charges. The mass error was set to 10 parts per million for precursor ions and 0.02 Da for fragment ions. Carbamidomethylation on cysteine was defined as a fixed modification and oxidation on methionine, acetylation on Lys, and acetylation at the protein N-terminus were specified as variable modifications. The false discovery rate threshold for protein, peptide, and modification site was specified as 1% (Fang et al., 2015; He et al., 2016). The minimum peptide length was set at seven amino acids. All of the other parameters in the MaxQuant analysis were set to default values. The probability of the identification of Lys acetylation site localization was set at >0.75 (Liu et al., 2016; Zhang et al., 2016).

### Bioinformatics analysis

#### Protein annotation, functional classification, and enrichment analysis

We used peptides identified from the proteome dataset of *P. asperata* somatic embryos during PDT (Jing et al., 2016) to validate the acetylated peptides we have found. The validated acetylated peptides were used to conduct the detail bioinformatics analysis. Characterization of the acetylated proteins was carried out using the Gene Ontology (GO) annotation according to biological process, cellular component, and molecular function terms. The annotation was derived from the UniProt-GOA database. When identified lysine-acetylated proteins were not annotated by the UniProt-GOA database, InterProScan software was used to describe the GO annotation of the protein based on the protein sequence alignment method (Dimmer et al., 2012). Kyoto Encyclopedia of Genes and Genomes (KEGG) annotation, domain description, and subcellular localization prediction were performed *via* Web-based interfaces and services, along with the bioinformatics tools, including KAAS (Moriya et al., 2007), KEGG mapper, InterProScan, and WoLF PSORT (PSORT/PSORT II) (Horton et al., 2007).

Functional enrichment analyses of GO terms (biological processes, cellular components, and molecular functions), KEGG pathways, and protein domains among the members of the resulting protein clusters were assessed using Fisher's exact test (two-tailed). For each category, the enrichment or depletion of the validated protein against all database proteins was tested. Correction for multiple hypothesis testing was carried out using the standard false discovery rate control method proposed by Benjamini and Hochberg. The annotation categories with adjusted *P*-values < 0.05 were considered as significant in all clusters (Huang et al., 2009). Finally, the filtered *P*-value matrix was transformed by the function x = −log10 (*P*-value), and the transformed x-values were used to construct figures. In addition, the selected pathways were classified into hierarchical categories according to the KEGG website, and the results were presented using R project “ggplot2” (http://cran.r-project.org/web/packages/ggplot2/).

#### Analysis of a model of sequences around acetylation sites

The model of the sequences comprising the ten amino acids upstream and downstream surrounding the LysAc sites in all acetylated proteins was analyzed using Soft Motif-X, as described in previous studies (Schwartz and Gygi, 2005; Chou and Schwartz, 2011; Fang et al., 2015). All of the database protein sequences were used as the background database parameters, and other parameters were set to the default.

#### Enrichment-based clustering analysis

After enrichment, all of the LysAc substrate categories obtained were collated based on *P*-values and then filtered for categories that were enriched in at least one of the clusters with a *P*-value of < 0.05. The filtered *P*-value matrix was then transformed by the function x = −log10 (*P*-value). Finally, the x-values obtained were z-transformed for every category, and the z scores were clustered *via* one-way hierarchical clustering (Euclidean distance, average linkage clustering) in Genesis (Sturn et al., 2002). All cluster memberships were visualized using a heat map from the “heatmap.2” function item of the “gplots” R-package (http://cran.r-project.org/web/packages/gplots/) (Wu et al., 2013).

#### Protein-protein interaction analysis

Protein-protein interaction (PPI) analysis was performed as in previous studies (Pan et al., 2014; Fang et al., 2015). Briefly, all name identifiers of the acetylated proteins were searched against the STRING database [version 9.1, (Szklarczyk et al., 2011)] for PPI. Only interactions between the proteins belonging to the searched dataset were selected, thereby excluding external candidates. STRING defines a metric known as a “confidence score” to define interaction confidence; we fetched all interactions with a confidence score ≥0.7 (high confidence). The interaction network from STRING was visualized using Cytoscape software (Shannon et al., 2003). A graph theoretical clustering algorithm [i.e., a molecular complex detection (MCODE) algorithm] was used to analyze densely connected regions.

## Results and discussion

### Identification of lysine-acetylated proteins in desiccated *P. asperata* somatic embryos

**Figure 1:**
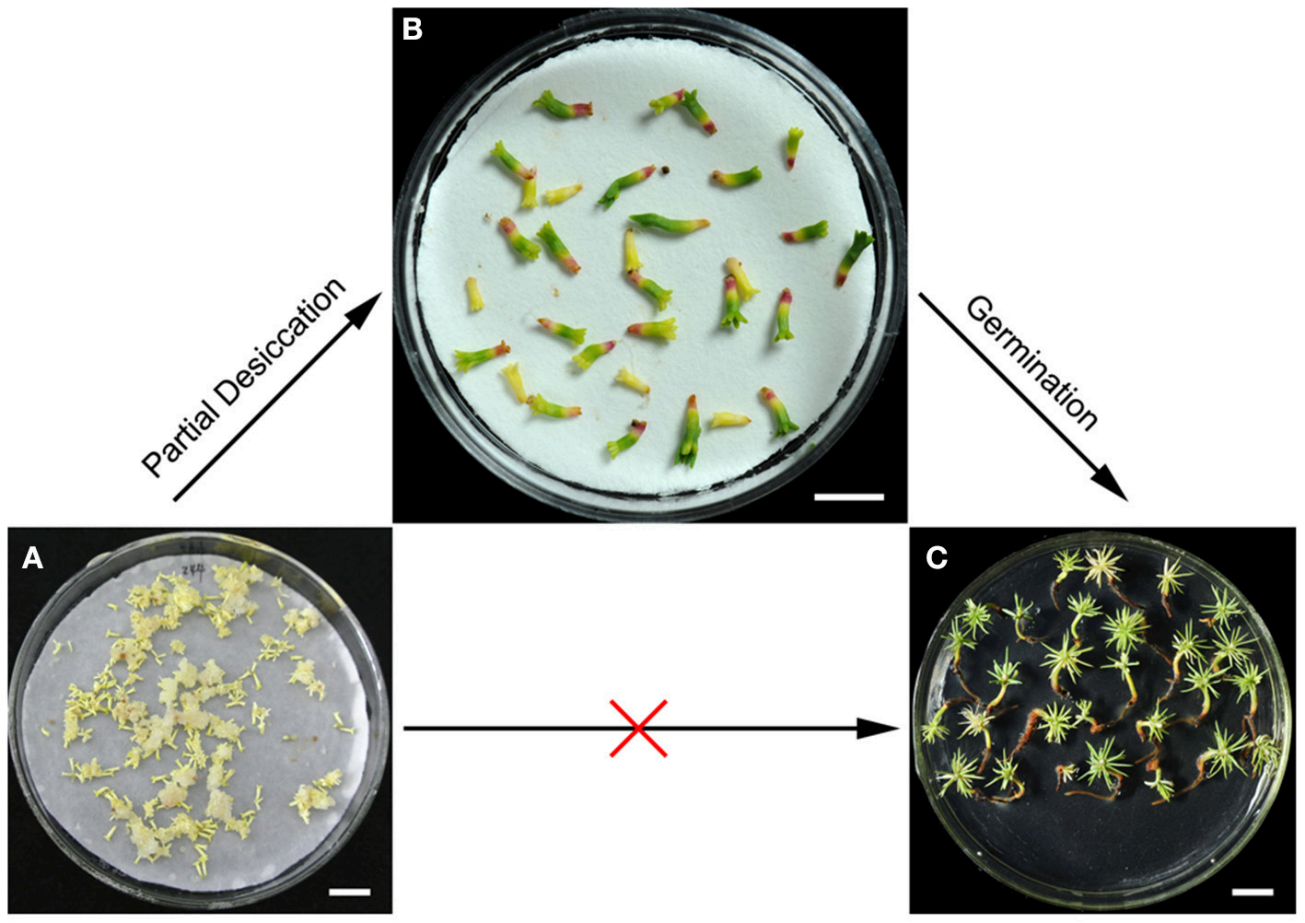
**Effects of partial desiccation on the morphology and germination of ***P. asperata*** somatic embryos**. Mature somatic embryos **(A)**, bar 10 mm; desiccated somatic embryos **(B)**, bar 5 mm; and germinants **(C)**, bar 10 mm.

Somatic embryogenesis of conifers, which uses plant cell engineering to generate plantlets, is one of the most important biotechnologies. To date, somatic embryogenesis has been achieved in a variety of coniferous species (Klimaszewska et al., 2016). However, the low germination rate of mature somatic embryos is a major limitation that prevents the application of somatic embryogenesis for plant propagation on a large scale. In the present study, PDT was found to promote germination and enhanced the conversion of *P. asperata* somatic embryos (Figure 1). Furthermore, we investigated the global acetylome of the desiccated embryos, to provide a better understanding of its physiological functions and the stress tolerance mechanisms.

**Figure 2:**
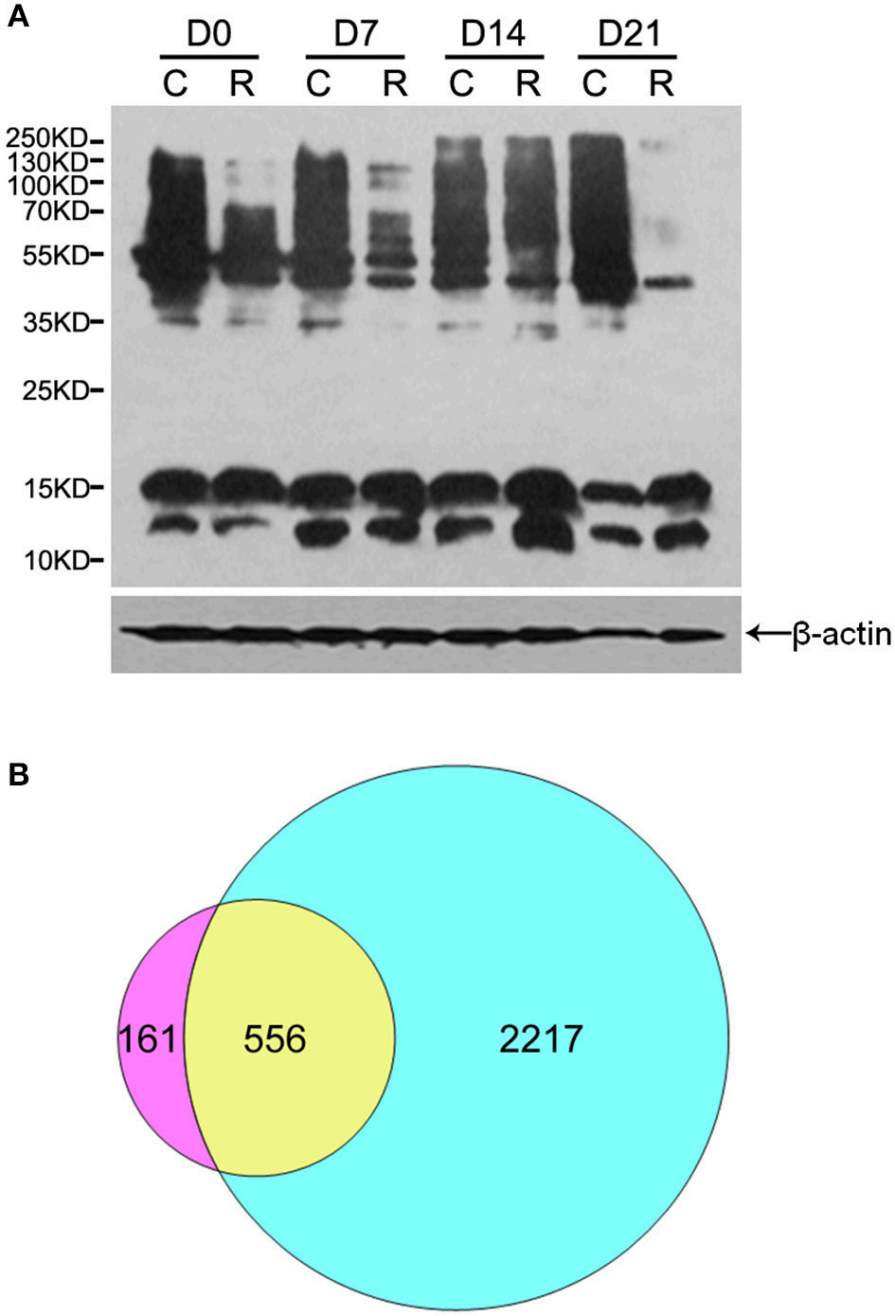
**Overview of acetylome analysis**. Western blotting analysis of proteins in the organs of somatic embryos using a pan anti-acetyllysine antibody. Cotyledons (C) and radicles (R) of somatic embryos during PDT. D0, D7, D14, and D21 indicate without PDT, and PDT for 7, 14, and 21 days, respectively **(A)**. Venn diagram shows the number of identified proteins unique to and common between lysine acetylome (left) and proteome (right, Jing et al., 2016) **(B)**.

To acquire a general view of LysAc in the embryos during PDT (D0, D7, D14, and D21), multiple major proteins bands with the molecular weights similar to histones and non-histones were successfully examined through western blotting using an anti-acetyllysine antibody (Figure 2A). Figure 2A shows that the level of LysAc was higher at D14. For example, the signals of 70–250 KD bands were enhanced upon anti-acetyllsine antibody detection in D14 radicles compared with D0, D7, and D21 radicles. On this basis, to obtain global insight into the large-scale dataset of LysAc sites in the desiccated embryos, we used antibody-based affinity enrichment and high-resolution MS to identify and analyze acetylated proteins in the embryos from D14.

The lengths of most identified peptides were distributed between 8 and 17 amino acids, which was consistent with tryptic peptide properties and MS identification (Supplementary Figure S1). In total, we successfully identified 1360 unique acetylation sites in 717 acetylated proteins in the desiccated embryos. The MS proteomics data have been deposited to the ProteomeXchange Consortium *via* the PRIDE (Vizcaino et al., 2016) partner repository with the dataset identifier PXD005042 (Username: Reviewer31026@ebi.ac.uk; Password: V62RrjWZ). By searching these acetylated peptides identified against the previous proteome dataset of the embryos during PDT, we validated 1079 unique acetylation sites in 556 acetylated proteins (Figure 2B and Supplementary Table S1). Previously, plant lysine acetylome studies identified 74 acetylated proteins in *A. thaliana* (Finkemeier et al., 2011), 31 in *S. tuberosum* (Xing and Poirier, 2012), 97 in *V. vinifera* (Melo-Braga et al., 2012), 121 in *G. max* (Smith-Hammond et al., 2014b), 358 in *P. sativum* (Smith-Hammond et al., 2014a), 684 in *F. ananassa* (Fang et al., 2015), 227 in *T. aestivum* (Zhang et al., 2016), and 716 in *O. sativa* (Xiong et al., 2016). These different numbers are mainly due to the different intrinsic acetylation levels of the proteins among the various species. Here, our data extend the inventory of lysine-acetylated proteins in plants, providing information to reveal the role of LysAc in conifer embryos as well as their development.

### Analysis of the distribution and conservation of LysAc sites

According to the validated 1079 LysAc sites in the 556 acetylated proteins, the average degree of acetylation was 1.9 sites per protein, and the number of LysAc sites in each protein was ranged from 1 to 9 (Supplementary Figure S2). Among these proteins, 57% contained only one acetylation site, and the percentages of proteins with two, three, four, and five or more modification sites were 20, 10, 7, and 6%, respectively. For the acetylome, the mean number of modified sites per 100 amino acids was 0.78, with the highest value being 6.67 (Supplementary Table S2).

To determine the evolutionary conservation of the detected LysAc sites, we searched the 1079 acetylated peptides in *P. asperata* against the previously reported acetylomes of some other species: *F*. *ananassa* (Fang et al., 2015), *O. sativa* (He et al., 2016; Xiong et al., 2016), and *A. thaliana* (Finkemeier et al., 2011; Wu et al., 2011). The consistency analysis demonstrated that 65 peptides from 51 proteins had been identified in at least two plants (Supplementary Table S3). Among these, 33 conserved proteins were involved in metabolic processes, indicating key functions of LysAc in metabolic regulation. Notably, seven metabolism-related proteins were conservatively acetylated at two sites among various plants species, including chalcone synthase (CHS, MA_10426264g0020), large subunit ribosomal protein L3e (RP-L3e, MA_10432758g0010), adenosylhomocysteinase (ahcY, MA_10433454g0020), small subunit ribosomal protein SAe (RP-SAe, MA_112104g0010), large subunit ribosomal protein L19e (RP-L19e, MA_1839g0010), elongation factor 1-alpha (EEF1A, MA_434977g0010), and phosphoglycerate kinase (PGK, MA_92421g0010) (Supplementary Table S3).

### Analysis of LysAc motifs in desiccated *P. asperata* somatic embryos

**Figure 3:**
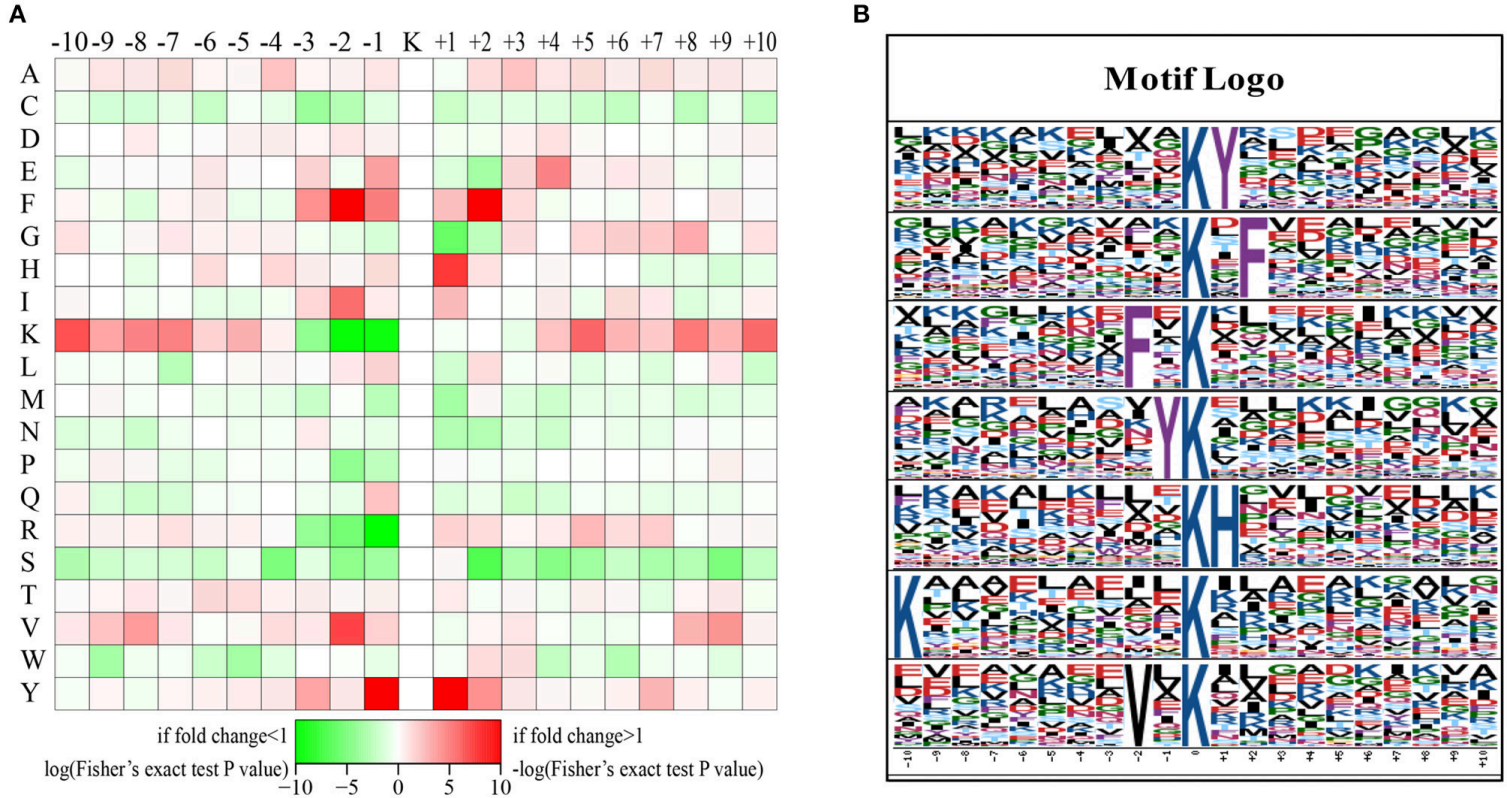
**Sequence motifs returned by Motif-X (motif length = 21, ten amino acids upstream and downstream of the acetylation site)**. Heat map showing over-representation of amino acid residues at positions from −10 to +10 from the acetylated lysine residue relative to the overall proteome background distribution **(A)**. Sequence logos for acetylation sites detected in proteins **(B)**.

The preferred amino acid residues surrounding LysAc sites have been identified in plants (Finkemeier et al., 2011; Melo-Braga et al., 2012; Nallamilli et al., 2014; Smith-Hammond et al., 2014a,b; Fang et al., 2015; He et al., 2016; Xiong et al., 2016; Zhang et al., 2016). To explore the conserved LysAc motifs of the desiccated somatic embryos of *P. asperata*, we used the Motif-X program to search for conserved sequence motifs in all of the validated 1079 acetylated-lysine sites. A total of 529 defined unique sites, accounting for 49% of the validated sites, were matched to seven conserved motifs (Figure 3 and Supplementary Table S1). Inspection of these seven motifs demonstrated that two types of amino acid residues surround acetylated sites: The first is phenylalanine (F), tyrosine (Y), lysine (K), or valine (V) upstream of the LysAc site, and the second is Y, F, or histidine (H) downstream of LysAc sites (Figure 3B). F and Y are two of the most conserved amino acid residues were detected both upstream and downstream of LysAc sites. F was located at the ±2 positions of the sites, while Y was detected at the ±1 positions. By contrast, the above two conserved residues (F and Y) in our dataset were the same as those in rice. In germinating rice seeds, F and Y are present at a high frequency around acetylated lysines (He et al., 2016). Xiong et al. (2016) found that F was an essential amino acid and was the most conserved residue surrounding the ±2 positions of LysAc sites in rice seedlings. Zhang et al. (2016) noted that H is preferred at the +1 position of LysAc sites, this is in accordance with our result. In addition, a residue with hydrophobic side chain group (V) was enriched at the −2 position. The above results suggested that these conserved motifs, which occur at the ±1 or ±2 positions of LysAc sites, are important for lysine acetylation in plants. Furthermore, a positively charged residue (K) was also found to be enriched at the −10 position in our study. These conserved amino acid residues surrounding LysAc sites suggest that residues with aromatic ring, positive charge or hydrophobic side chain might be functionally important for acetylation to occur.

### Functional characterization of lysine-acetylated proteins in desiccated *P. asperata* somatic embryos

**Figure 4:**
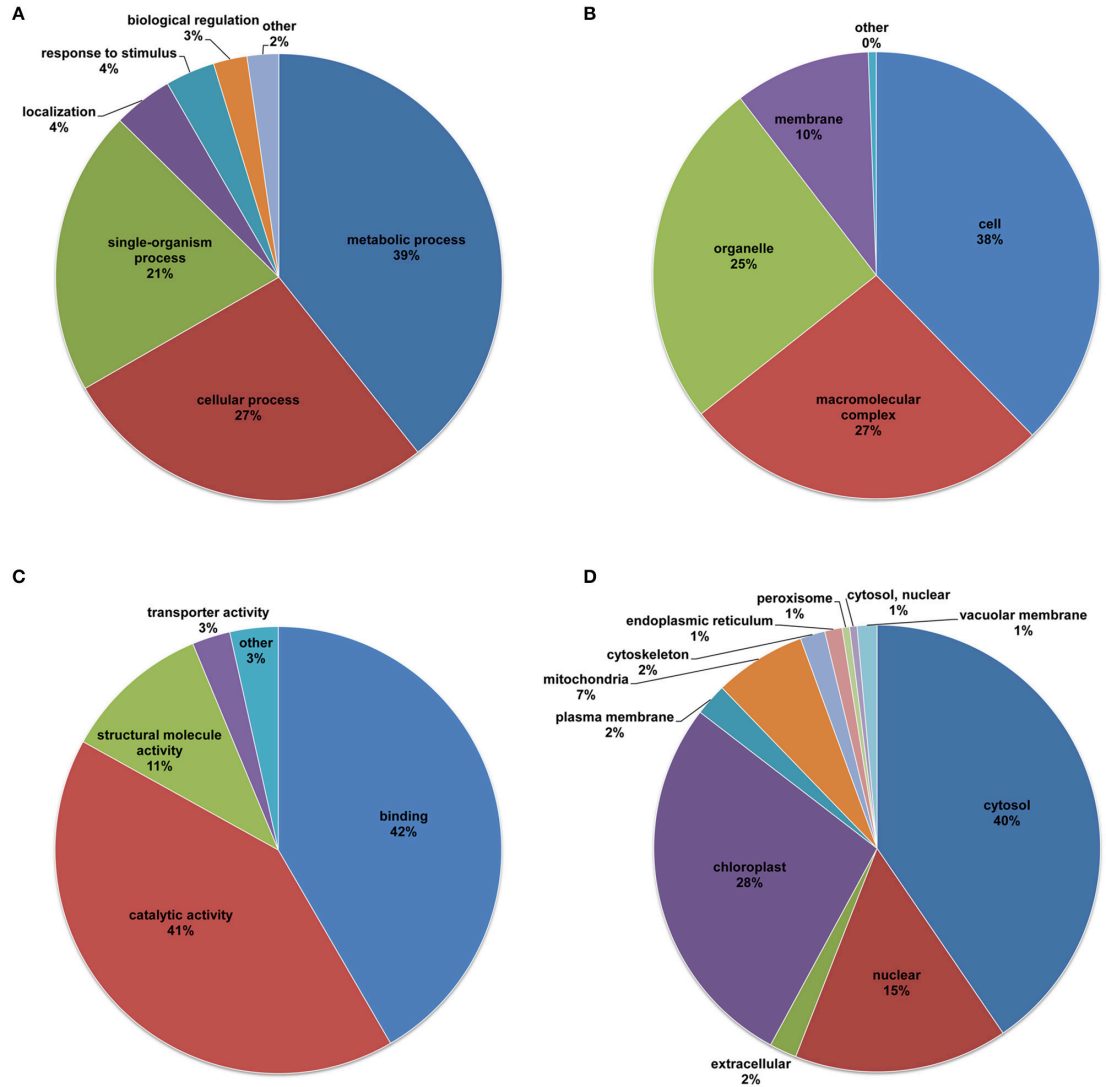
**Distribution of all of the lysine-acetylated proteins of the desiccated embryos**. GO categories of biological processes **(A)**, cellular components **(B)**, and molecular functions **(C)**, and subcellular location predictions **(D)**.

To better understand the function of the lysine acetylome in desiccated embryos, the lysine-acetylated proteins were subjected to GO classification based on the corresponding biological processes, cellular components, and molecular functions (Figure 4 and Supplementary Table S4). In the functional classification of biological processes, the acetylated proteins of the desiccated embryos were involved in metabolic processes, cellular processes, and single-organism processes, accounting for 39, 27, and 21% of all lysine-acetylated proteins, respectively (Figure 4A). Similarly, previous studies have indicated that proteins involved in many metabolic and cellular processes can be acetylated at lysine sites. In addition, this reversible lysine acetylation is crucial for the regulation of biological processes in *A. thaliana* (Wu et al., 2011), *F. ananassa* (Fang et al., 2015), and *O. sativa* (He et al., 2016; Xiong et al., 2016). These lysine-acetylated proteins validated in the present study could be important for conifer metabolism and cellular regulation. Furthermore, the percentage of acetylated proteins in the desiccated embryos in the response to stimulus category was slightly higher than that in the non-desiccated embryos (Supplementary Figure S3A), implying an important regulatory role of lysine acetylation in the response of embryos to desiccation stress.

For the cellular component ontology, the acetylated proteins were mainly classified into the cell (38%), macromolecular complex (27%), organelle (25%), and membrane (10%) categories (Figure 4B). It has been reported that the main cellular components of rice are cells, organelles, membranes, and macromolecular complexes, accounting for 29, 23, 17, and 11% of all acetylated proteins, respectively (He et al., 2016). In strawberry leaves, the top three cellular components are organelles (55.66%), macromolecular complexes (24.53%), and membranes (18.92%) (Fang et al., 2015). Based on these data, we speculate that the main classifications of cellular components are essentially the same among acetylated plant proteins.

In the ontology of molecular functions, the analysis demonstrated that the acetylated proteins were associated with binding (42%), catalytic activity (41%), and structural molecular activity (11%) (Figure 4C). The percentage of acetylated proteins in the catalytic activity category was much higher in desiccated embryos than in non-desiccated embryos (Supplementary Figure S3C). It is well known that the catalytic activity of enzymes involves the binding of their substrates to form an enzyme-substrate complex (Cooper, 2000). Previous reports revealed that reversible lysine acetylation in the binding domain regulates metabolism to respond to different stressors in bacteria (Mo et al., 2015; Liu et al., 2016). In the present, we infer that the majority of the validated acetylated proteins, which are transcription factors or enzyme-related proteins, ensured that the embryos responded promptly to desiccation stress.

To reveal the subcellular localization of acetylation in the desiccated embryos, we conducted subcellular location prediction (Figure 4D and Supplementary Table S4). The prediction indicated that the majority of the validated acetylated proteins were distributed in diverse subcellular locations with 40% in cytosol, suggesting that LysAc in this compartment plays an important role in the embryos. Furthermore, a number of acetylated proteins were also localized to the chloroplasts (28%), nuclei (15%), and mitochondria (7%). Due to the different materials examined, our data differed slightly from other plant systems such as strawberry, rice, and *Arabidopsis*. Fang et al. (2015) demonstrated that the main subcellular components of strawberry leaves are chloroplasts, cytoplasmic components and nuclei, accounting for 48, 28, and 7% of all acetylated proteins, respectively. In whole rice plants, the main subcellular components are chloroplasts (42%), cytoplasm (29%), nuclei (12%), and mitochondria (8%) (Xiong et al., 2016). The subcellular components of *Arabidopsis* leaves mainly correspond to the intracellular (23.1%), cytoplasm (15.7%), and chloroplasts (14.4%) categories (Wu et al., 2011). Notably, the percentage of acetylated proteins in the chloroplasts was higher in the desiccated embryos than in the non-desiccated embryos (Supplementary Figure S3D), which was directly associated with the low-light conditions, indicating that the embryos carried out photosynthesis under light in response to nutrient-deficit stress. In addition, 1% of the acetylated proteins in the desiccated embryos were localized to the peroxisome, where fatty acids are activated to acyl-CoA and then converted to sucrose (Rylott et al., 2006).

**Figure 5:**
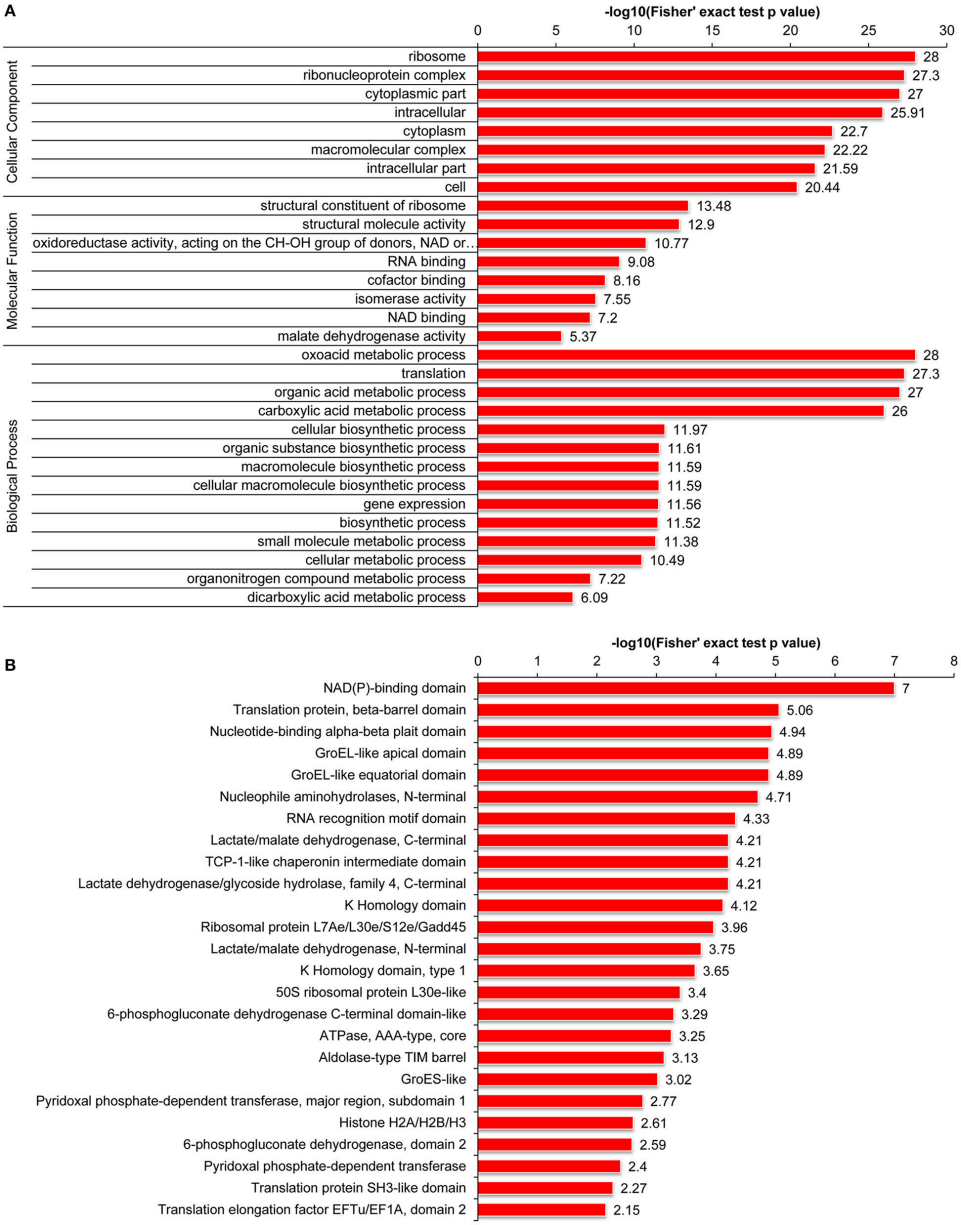
**Enrichment of acetylated proteins in the desiccated embryos**. GO enrichment analysis of acetylated proteins, including cellular components, molecular functions, and biological processes **(A)**. Protein domain enrichment analysis of acetylated proteins **(B)**. Every annotation is presented in comparison with the corresponding percentage annotation values for the whole genome. The hypergeometric test with Bejamini and Hochberg false discovery rate correction was used for statistical analysis, and the significance cutoff was *P* <0.05.

Furthermore, we carried out GO enrichment analysis to determine which functional terms were targeted for LysAc in the desiccated embryos (Figure 5A). In the analysis of cellular components, ribosome and ribonucleoprotein complexes were significantly enriched. Accordingly, the structural constituents of the ribosome were significantly enriched in the molecular function category. The detailed enrichment results are presented in Supplementary Table S5. Notably, oxidoreductase activities were significantly enriched in the desiccated embryos compared with those in the non-desiccated embryos (Supplementary Figure S4A), reflecting a crucial role of LysAc in the response to desiccation stress. Our data also demonstrated significant enrichment of validated proteins in various biological processes, including the oxoacid metabolic processes, organic acid metabolic processes, and carboxylic acid metabolic processes, indicating the pivotal role of LysAc in virtually all fundamental metabolic processes.

Furthermore, to search for enriched protein domains in the acetylated proteins of *P. asperata*, we applied protein domain enrichment analysis to the acetylome data. In total, 25 protein domains were significantly enriched (Figure 5B and Supplementary Table S5). The validated proteins with these enriched domains are involved in various metabolic pathways with important cellular functions. Specifically, the NAD(P)-binding domain was significantly enriched in the desiccated embryos compared with the non-desiccated embryos (Supplementary Figure S4B). A previous study suggested that enzymes containing an NAD(P)-binding domain are involved in catalyzing redox reactions (Hua et al., 2014). This further supports the hypothesis that LysAc plays a key role in the response to desiccation stress.

### Lysine-acetylated proteins are involved in the stress-related response

On the first day of PDT, the embryos were subjected to severe water and nutrient deficiency, leading to a series of physiological changes (Jing et al., 2016). The initial slow desiccation is thought to be the signal that triggers events that establish desiccation tolerance, embryo regeneration, and subsequent germination (Lipavská and Konrádová, 2004). In the subsequent days of PDT, with the gradually increased water content, the embryos exhibited shoot and root growth (Figure 1B). Additionally, somatic embryos after PDT germinated effectively and vigorously (Figure 1C).

Previous studies on plants have indicated that LysAc plays important roles in many cellular processes, including responses to changes in environmental conditions, shoot and root development, and hormone signaling (Chen and Tian, 2007; Servet et al., 2010). In the desiccated embryos, we detected three acetylated proteins that were located in the peroxisome (Supplementary Table S4), which is associated with the reduction of reactive oxygen species, such as hydrogen peroxide (Bonekamp et al., 2009) and the degradation of fatty acids (Rylott et al., 2006). Two pivotal enzymes, catalase (MA_10437148g0010 and MA_10427891g0010) and malate synthase (MA_120902g0010), were found to be modified at multiple sites by LysAc in the desiccated embryos (Supplementary Table S1) compared with the non-desiccated embryos. Additionally, the analysis of biological processes indicated that in the desiccated embryos, 25 acetylated proteins were enriched in response to stimulus-related GO terms (Supplementary Table S4). Some peroxidases, such as glutathione peroxidase (MA_125131g0010), cationic peroxidase SPC4 (MA_8813326g0010), and probable L-ascorbate peroxidase 8 (MA_119996g0010) (Supplementary Table S1), were not detected in non-desiccated embryos.

### Lysine-acetylated proteins involved in central metabolism

**Figure 6:**
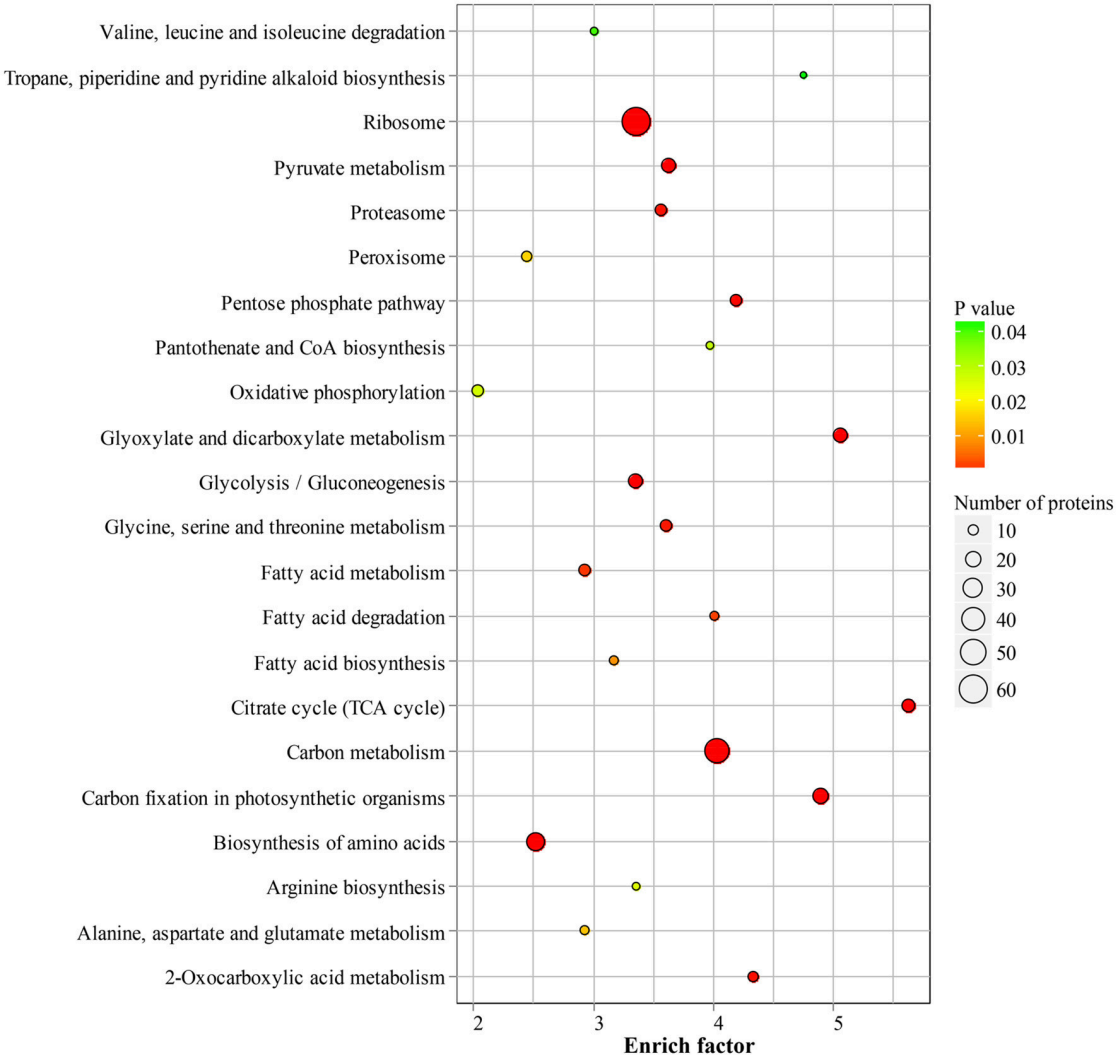
**Significantly enriched KEGG pathways of all of the lysine-acetylated proteins in the desiccated embryos**. Every annotation is presented in comparison with the corresponding percentage annotation values for the whole genome. The hypergeometric test with Bejamini and Hochberg false discovery rate correction was used for statistical analysis, and the significance cutoff was *P* < 0.05.

To reveal the preferential metabolic pathways associated with LysAc in the desiccated embryos, we performed KEGG pathway enrichment analysis. Cluster analysis showed that 251 acetylated candidate proteins revealed involvement in 22 enriched pathways (Figure 6, Supplementary Table S5 and Supplementary Package 1), among which carbon metabolism was significantly enriched. It is well known that glycolysis/gluconeogenesis and the tricarboxylic acid cycle are the main pathways of glycometabolism and denote the diverse biochemical processes of various carbohydrates in living cells (Plaxton, 1996; Mo et al., 2015; He et al., 2016). It has also been reported that acetylation of glycometabolism-related proteins (mainly enzymes) may be a crucial regulatory mechanism in processes such as plant development, growth, and stress tolerance (Fang et al., 2015; He et al., 2016). In our study, a large proportion of the enzymes in these two pathways were detected as being acetylated both in desiccated and non-desiccated embryos (Supplementary Figure S5), which facilitates the elucidation of the mechanism of LysAc-regulated glycometabolism. We infer that the lysine-acetylated residues of key enzymes are a prerequisite for regulating the glycometabolic pathway in living organisms.

**Figure 7:**
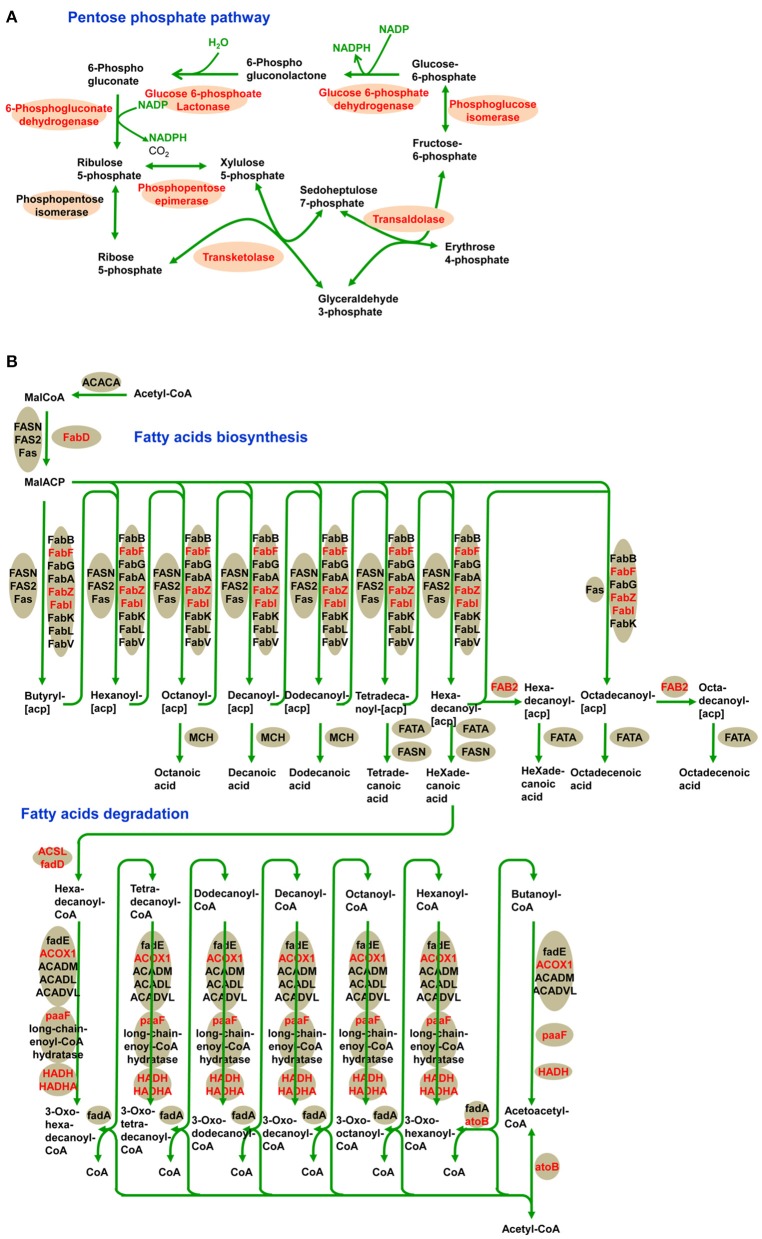
**Acetylation of central metabolic enzymes in desiccated embryos**. Pentose phosphate pathway **(A)** and fatty acids metabolism **(B)**. The characters in red show acetylated enzymes.

In addition, we identified a significantly enriched pathway of pentose phosphate in the desiccated embryos (Figure 7A and Supplementary Table S5). The pentose phosphate pathway is considered to be a major source of cellular reducing power and plays an essential protective role against oxidative stress (Kletzien et al., 1994; Pandolfi et al., 1995). Glucose-6-phosphate dehydrogenase (MA_10267514g0010) catalyzes the first step in the pentose phosphate pathway and provides reductive potential in the form of NADPH (Slekar et al., 1996). The production of NADPH in turn supports antioxidant enzyme activity (Lehmann et al., 2009). In the pentose phosphate pathway, we infer that LysAc plays an important role in protecting the biomolecules of the desiccated embryos from oxidative damage.

Fatty acid metabolism was enriched in desiccated embryos compared with that in non-desiccated embryos (Figure 7B and Supplementary Table S5). Fatty acid degradation mainly proceeds through β-oxidation, which provides respiratory substrates under conditions of carbohydrate starvation (Dieuaide et al., 1992), produces hydrogen peroxide that may act as a signal in various stress responses in plants (Eastmond and Graham, 2000), and supplies metabolic energy and carbon skeletons for germination (Rylott et al., 2006). These functions are essential in the PDT process, not only in the response to water deficiency but also in supporting growth until photosynthetic competence is achieved.

### Functional enrichment based on clustering analysis

**Figure 8:**
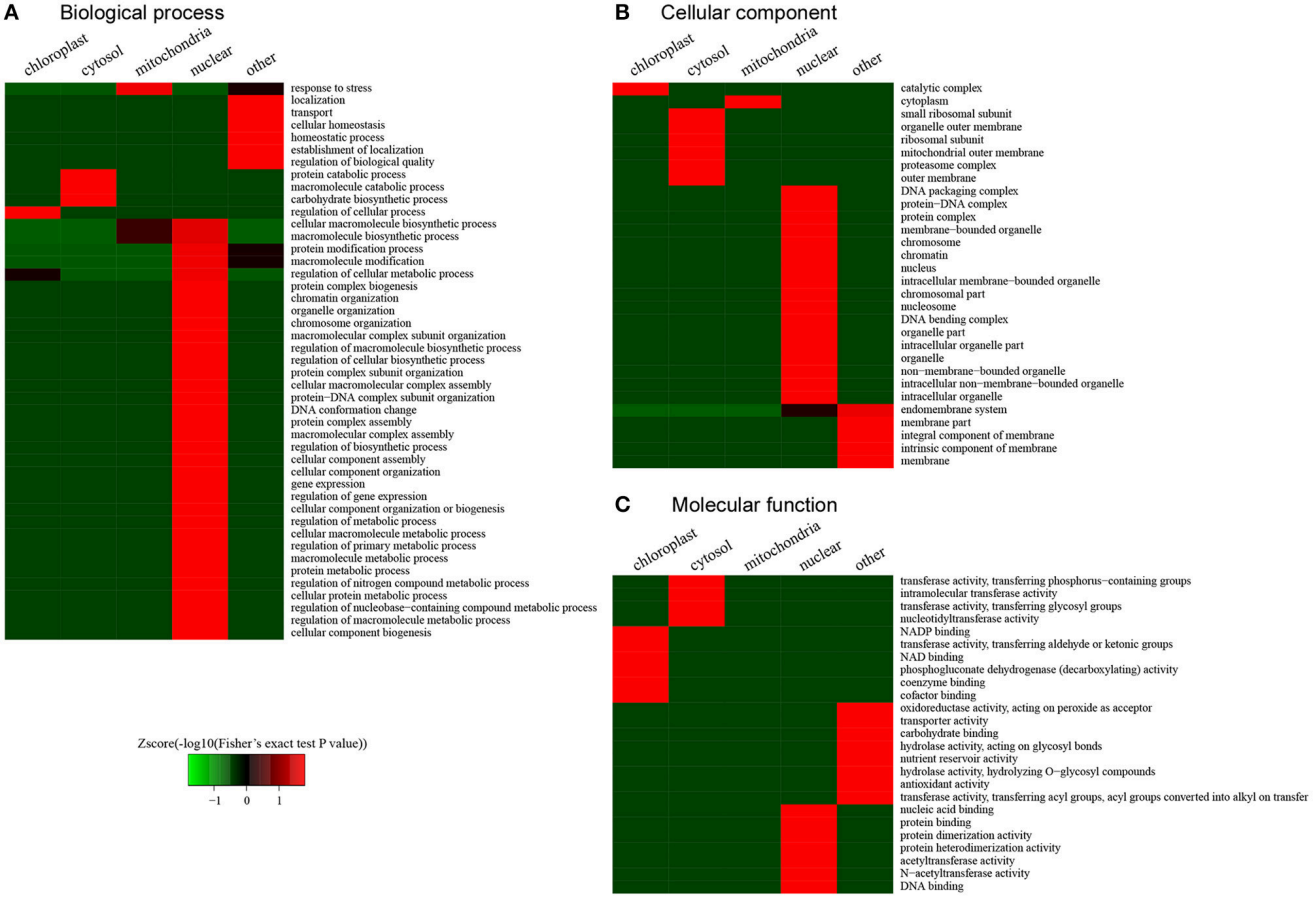
**Functional clustering analysis of all of the lysine-acetylated proteins in the desiccated embryos**. GO categories of biological processes **(A)**, cellular comsponents **(B)**, and molecular functions **(C)**.

According to the clustering analysis of subcellular locations in the desiccated embryos, we conducted functional enrichment to characterize the preferential target substrates of LysAc in different organelles (Figure 8). The response to stress was significantly enriched in mitochondria according to the biological processes category (Figure 8A). This enrichment indicated that during PDT, stress-related proteins were the preferential target substrates of LysAc in mitochondria. Accordingly, the molecular function category demonstrated that stress-related GO classifications, oxidoreductase, nutrient reservoir, and antioxidant activities, were significantly enriched (Figure 8C). In the nucleus, many chromatin organization-, protein complex-, protein-DNA complex-, and DNA conformation change-related classifications were significantly enriched (Figure 8A). Accordingly, many DNA, protein complex, protein-DNA complex, and chromatin classification in the cellular component category were significantly enriched in the nucleus (Figure 8B). The enrichment of nucleic acid, protein and DNA binding, and protein dimerization and heterodimerization activity was also significant in the nucleus in the molecular functions category (Figure 8C). In chloroplasts, the enzyme catalytic complex was significantly enriched (Figure 8B), suggesting that LysAc mainly occurs in catalytic metabolism-related proteins in this organelle. Consistent with this enrichment, several enzymes-related factors such as NADP, NAD, coenzymes, and cofactor binding, were significantly enriched in the chloroplasts, based on the classification of molecular functions (Figure 8C).

These differential clustering classifications in different organelles may contribute to stress and the intrinsic subcellular location of LysAc. First, during PDT, somatic embryos of *P. asperata* suffer from a water-deficit, nutrient-deficit, and oxidative stress. Thus, we looked for the acetylated proteins in response to stress, and the clustering analysis revealed that the stress-related GO classifications, including oxidoreductase activity, hydrolase activity, nutrient reservoir activity, and antioxidant activity, were significantly enriched. Furthermore, chromatin organization- and DNA conformation-related categories were significantly enriched in the nucleus. Previous studies have shown that the LysAc of histones is involved in chromatin structure, histone-DNA, and histone-histone interactions (Strahl and Allis, 2000; Chen and Tian, 2007; Hirschey et al., 2011).

### Protein-protein interaction (PPI) network analysis of lysine-acetylated proteins

**Figure 9:**
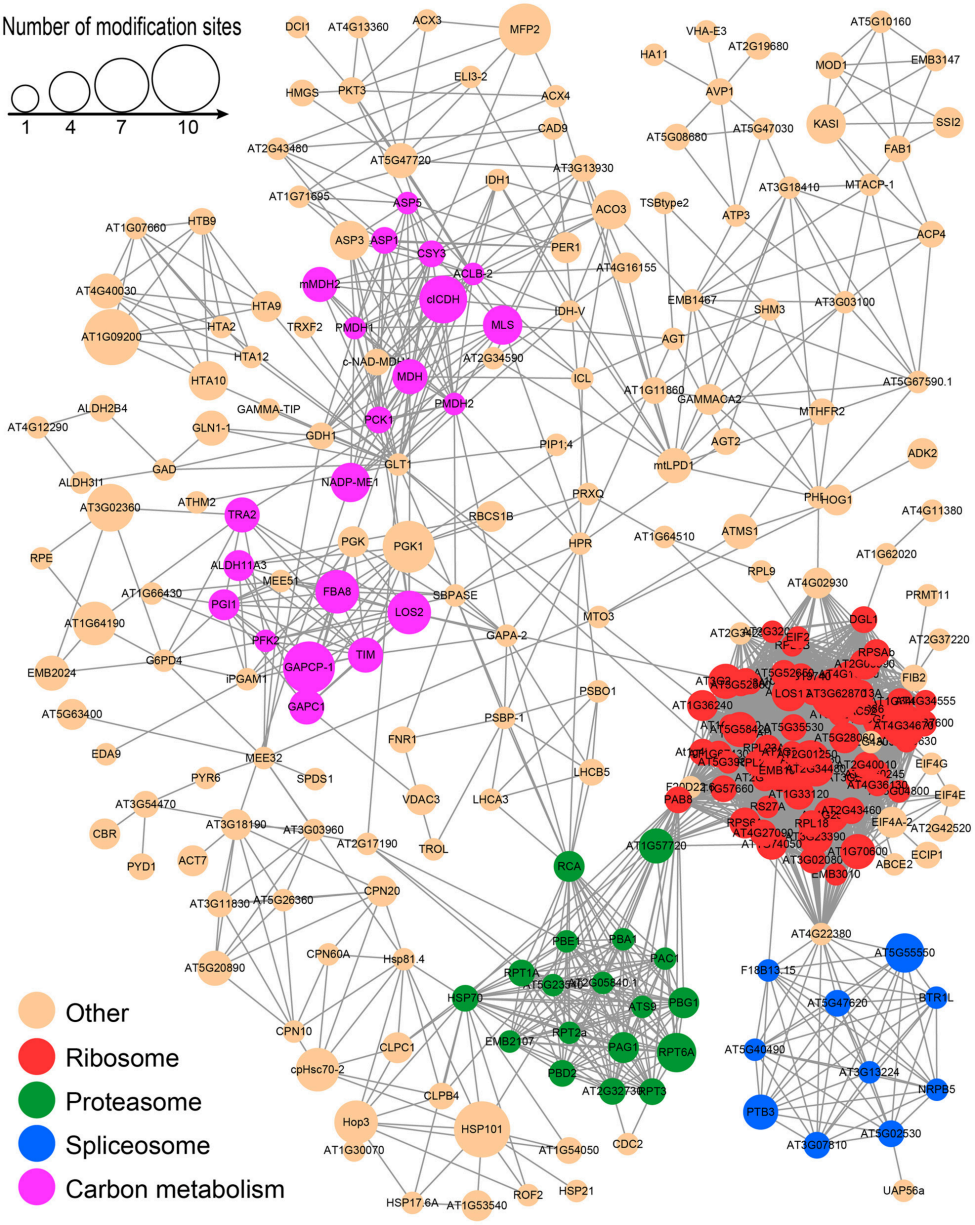
**PPI network of all of the lysine-acetylated proteins in the desiccated embryos**.

To better understand the cross-linked pathways of these acetylated proteins, we established a PPI network among the acetylated proteins of the desiccated embryos based on information from the STRING database (Figure 9 and Supplementary Table S6). An overview network of 257 acetylated proteins was constructed to represent the diverse functions in the desiccated embryos. Among these networks, four highly-connected clusters, including the ribosome, proteasome, spliceosome, and metabolites of carbon metabolism clusters, were extracted using the MCODE algorithm (Supplementary Figure S6). Most of these interacting proteins were associated with the ribosome, proteasome, and spliceosome clusters, which is in agreement with the analysis of subcellular location that was associated with the synthesis and degradation of stress-related proteins. In addition, many interacting acetylated proteins were also classified as part of carbon metabolism, suggesting that LysAc on glycometabolism-related proteins plays an important role for embryo development.

## Conclusion

In this study, using antibody-based affinity enrichment and high-resolution MS, we comprehensively investigated the lysine acetylome in *P. asperata* desiccated somatic embryos. We identified and validated 1079 acetylation sites in 556 acetylated proteins, thus expanding the lysine acetylome catalog in plants. Functional analysis of acetylated proteins revealed that LysAc is mainly involved in the response to stress and glycometabolism. Furthermore, the clustering analysis of subcellular locations suggested that lysine acetylation plays a key role in the response to stress under PDT. Our study provides new insights into the regulation of LysAc in *P. asperata* somatic embryos under PDT, and these findings may be applied to genetic engineering for high stress-tolerance in conifers.

## Author contributions

YX and DJ conducted the experiments and drafted the manuscript; LK advised on experimental design and provided language revision; JZ carried out tissue sample culturing; FO contributed to the data analysis; and HZ, JW, and SZ provided plant tissues, laboratory facilities, and project supervision. All authors approved the final draft of the manuscript.

## Funding

This study was supported by a grant from the China Twelfth Five-Year Plan for Science & Technology Support (2012BAD01B01).

### Conflict of interest statement

The authors declare that the research was conducted in the absence of any commercial or financial relationships that could be construed as a potential conflict of interest. The reviewer JK and handling Editor declared their shared affiliation, and the handling Editor states that the process nevertheless met the standards of a fair and objective review.

## Supplementary material

Supplementary Table S1 **List of all of the identified and validated acetylated sites and proteins in *P. asperata* desiccated somatic embryos**.

Supplementary Table S2 **Frequency of LysAc of proteins in *P. asperata* desiccated somatic embryos**. Statistical analysis was based on the number of modified sites per 100 amino acids for every validated protein.

Supplementary Table S3 **Conservation analysis of lysine-acetylated peptides in *P. asperata, Fragaria ananassa, Oryza sativa*, and *Arabidopsis thaliana***.

Supplementary Table S4 **GO and subcellular classifications of all lysine-acetylated proteins in *P. asperata* desiccated somatic embryos**.

Supplementary Table S5 **GO, KEGG, and protein domain enrichment of all lysine-acetylated proteins in *P. asperata* desiccated somatic embryos**.

Supplementary Table S6 **Lysine-acetylated proteins in the PPI network**.

Supplementary Figure S1 **Distribution of acetylated peptides based on length**.

Supplementary Figure S2 **Column diagram demonstrating the number of modified sites in a protein**.

Supplementary Figure S3 **Distribution of all of the lysine-acetylated proteins of the non-desiccated embryos**. GO categories of biological processes **(A)**, cellular components **(B)**, and molecular functions **(C)**, and subcellular location predictions **(D)**.

Supplementary Figure S4 **Enrichment of acetylated proteins in the non-desiccated embryos**. GO enrichment analysis of acetylated proteins, including cellular components, molecular functions, and biological processes **(A)**. Protein domain enrichment analysis of acetylated proteins **(B)**. Every annotation is presented in comparison with the corresponding percentage annotation values for the whole genome. The hypergeometric test with the Bejamini and Hochberg false discovery rate correction was used for statistical analysis, and the significance cutoff was *P* < 0.05.

Supplementary Figure S5 **Significantly enriched KEGG pathways for all of the lysine-acetylated proteins of the non-desiccated embryos**. Every annotation is presented in comparison with the corresponding percentage annotation values for the whole genome. The hypergeometric test with the Bejamini and Hochberg false discovery rate correction was used for statistical analysis, and the significance cutoff was *P* < 0.05.

Supplementary Figure S6 **Significantly enriched sub-clusters extracted from the whole PPI network**. Ribosome **(A)**, proteasome **(B)**, spliceosome **(C)**, and carbon metabolism **(D)**.

Supplementary Package 1 **Details of all of the significantly enriched KEGG pathways in *P. asperata* desiccated somatic embryos**.
