# Supplementary figures and images for: Global Lysine Acetylome Analysis of Desiccated Somatic Embryos of Picea asperata
Source: Front Plant Sci. 2016 Dec 23;7:1927. doi: 10.3389/fpls.2016.01927 (PMC5179564; doi:10.3389/fpls.2016.01927)

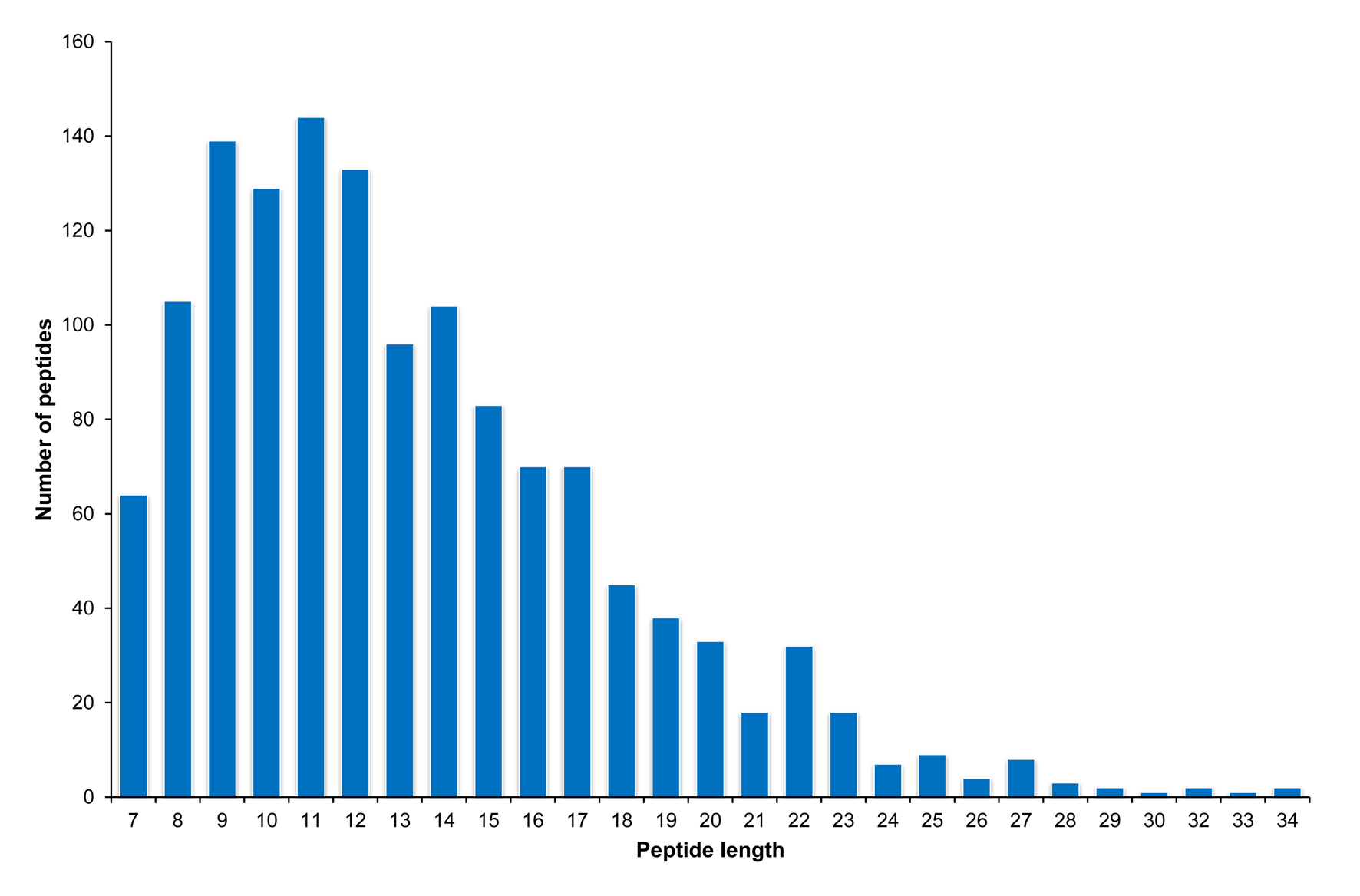

Supplement: Supplementary Figure S1 — Distribution of acetylated peptides based on length. [file Image1.TIF]

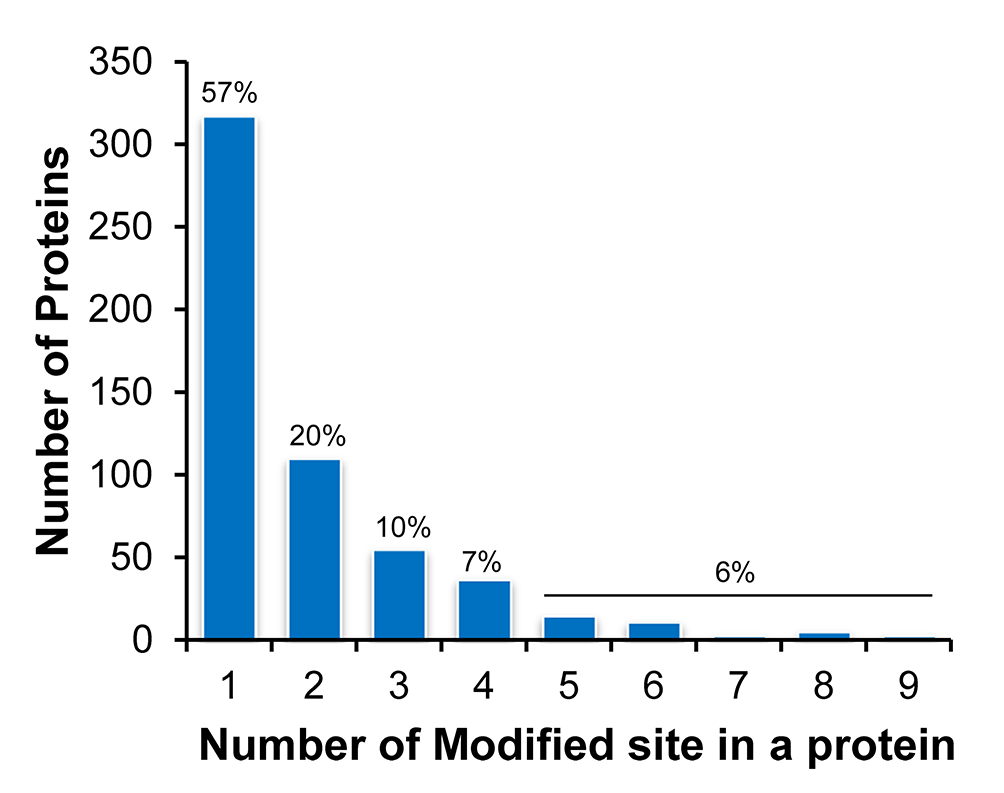

Supplement: Supplementary Figure S2 — Column diagram demonstrating the number of modified sites in a protein. [file Image2.TIF]

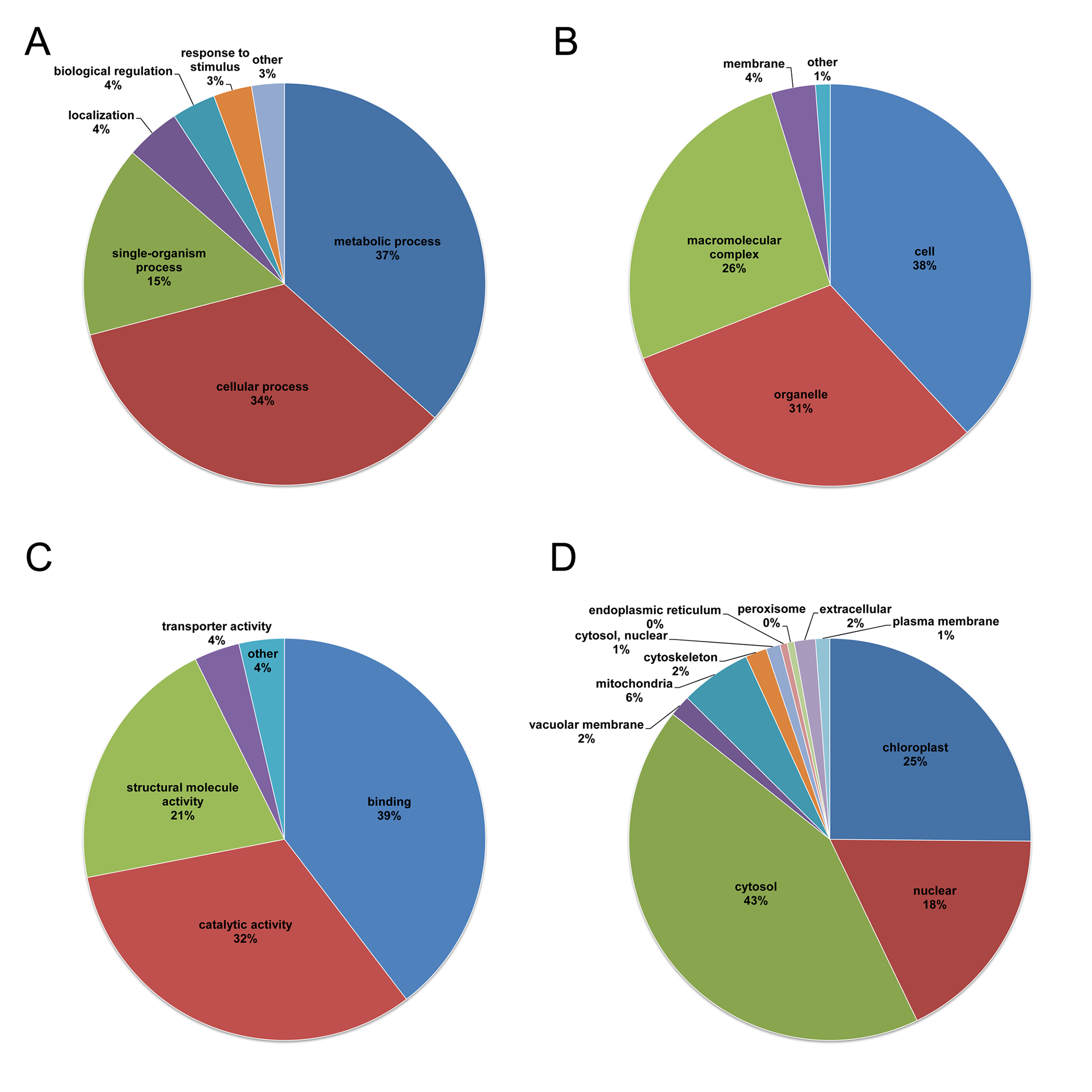

Supplement: Supplementary Figure S3 — Distribution of all of the lysine-acetylated proteins of the non-desiccated embryos. GO categories of biological processes (A), cellular components (B), and molecular functions (C), and subcellular location predictions (D). [file Image3.TIF]

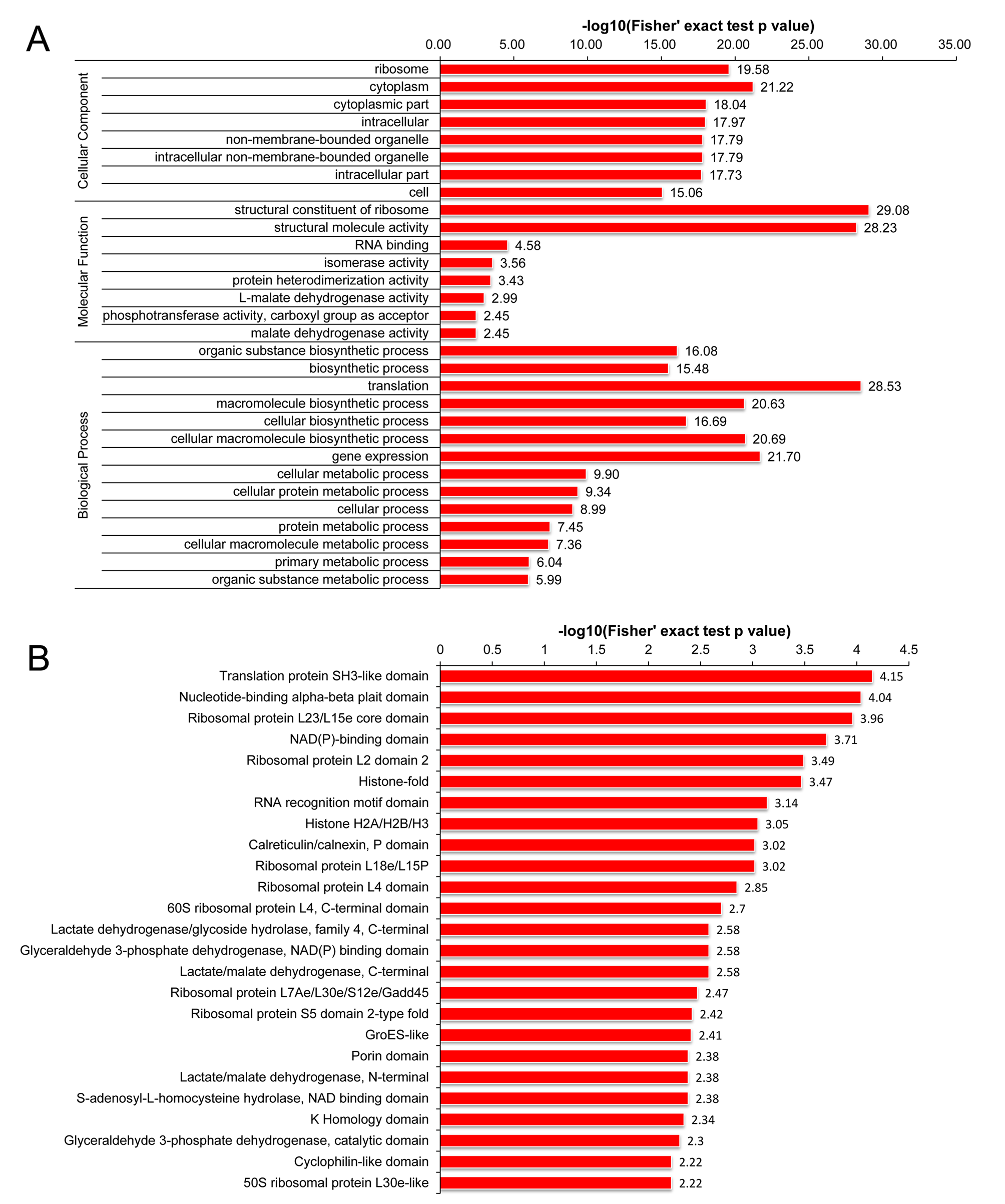

Supplement: Supplementary Figure S4 — Enrichment of acetylated proteins in the non-desiccated embryos. GO enrichment analysis of acetylated proteins, including cellular components, molecular functions, and biological processes (A). Protein domain enrichment analysis of acetylated proteins (B). Every annotation is presented in comparison with the corresponding percentage annotation values for the whole genome. The hypergeometric test with the Bejamini and Hochberg false discovery rate correction was used for statistical analysis, and the significance cutoff was P < 0.05. [file Image4.TIF]

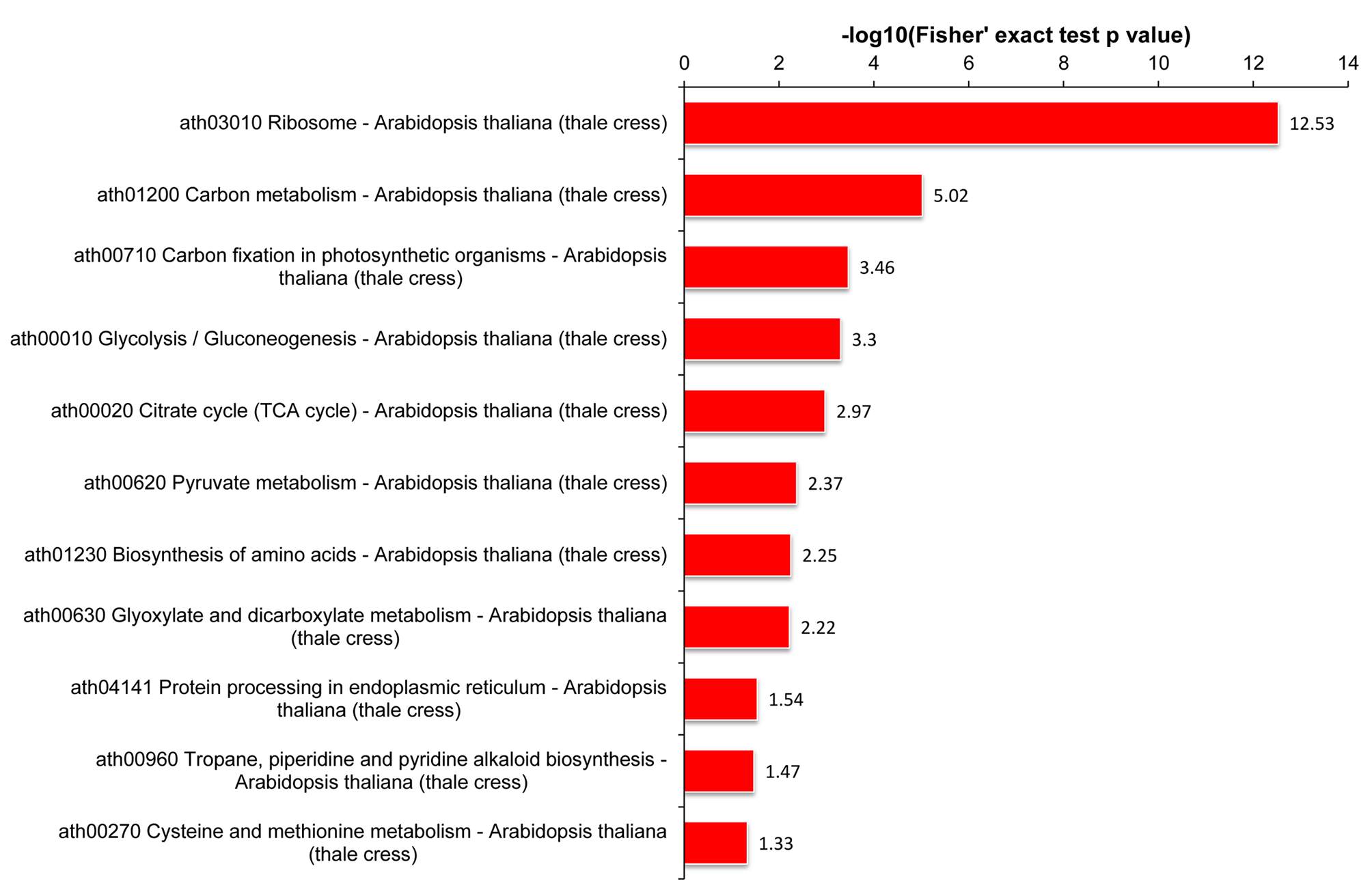

Supplement: Supplementary Figure S5 — Significantly enriched KEGG pathways for all of the lysine-acetylated proteins of the non-desiccated embryos. Every annotation is presented in comparison with the corresponding percentage annotation values for the whole genome. The hypergeometric test with the Bejamini and Hochberg false discovery rate correction was used for statistical analysis, and the significance cutoff was P < 0.05. [file Image5.TIF]

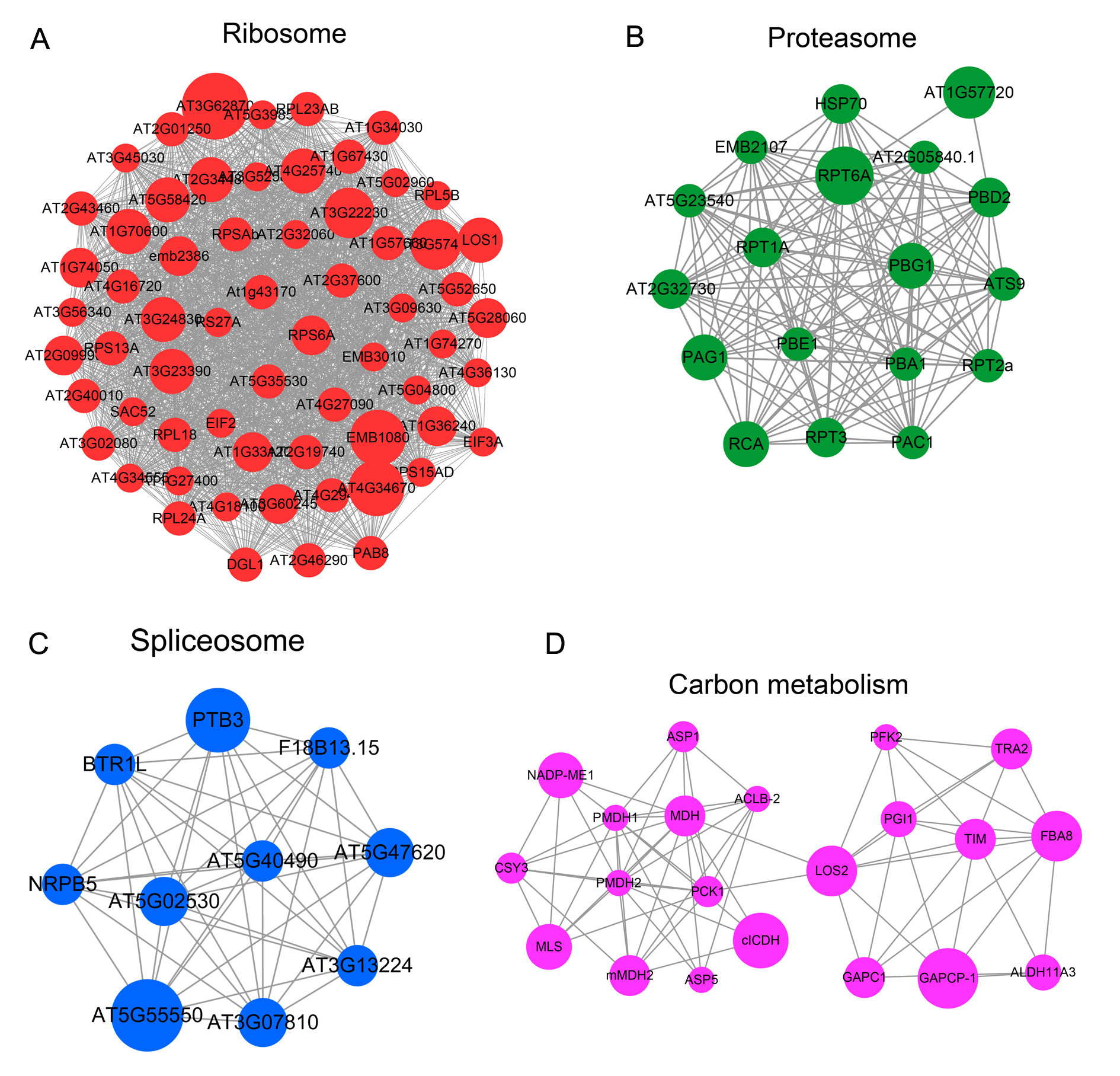

Supplement: Supplementary Figure S6 — Significantly enriched sub-clusters extracted from the whole PPI network. Ribosome (A), proteasome (B), spliceosome (C), and carbon metabolism (D). [file Image6.TIF]
